# Controlling a Random Population

**DOI:** 10.1007/978-3-030-45231-5_7

**Published:** 2020-04-17

**Authors:** Thomas Colcombet, Nathanaël Fijalkow, Pierre Ohlmann

**Affiliations:** 8 ENS/CNRS, Université Paris-Saclay, Cachan, France; 9 University of Duisburg-Essen, Duisburg, Germany; 10 Université de Paris, IRIF, CNRS, Paris, France; 11 CNRS, LaBRI, Bordeaux, France; 12 The Alan Turing Institute of Data Science, London, United Kingdom

## Abstract

Bertrand et al. introduced a model of parameterised systems, where each agent is represented by a finite state system, and studied the following control problem: for any number of agents, does there exist a controller able to bring all agents to a target state? They showed that the problem is decidable and **EXPTIME**-complete in the adversarial setting, and posed as an open problem the stochastic setting, where the agent is represented by a Markov decision process. In this paper, we show that the stochastic control problem is decidable. Our solution makes significant uses of well quasi orders, of the max-flow min-cut theorem, and of the theory of regular cost functions.

## 1 Introduction

*The control problem for populations of identical agents.* The model we study was introduced in [3] (see also the journal version [4]): a population of agents are controlled uniformly, meaning that the controller applies the same action to every agent. The agents are represented by a finite state system, the same for every agent. The key difficulty is that there is an arbitrary large number of agents: the control problem is whether for every $n \in \mathbb{N}$, there exists a controller able to bring all $n$ agents synchronously to a target state.

The technical contribution of [3,4] is to prove that in the adversarial setting where an opponent chooses the evolution of the agents, the (adversarial) control problem is **EXPTIME**-complete.

In this paper, we study the stochastic setting, where each agent evolves independently according to a probabilistic distribution, *i.e.* the finite state system modelling an agent is a Markov decision process. The control problem becomes whether for every $n \in \mathbb{N}$, there exists a controller able to bring all $n$ agents synchronously to a target state with probability one.

* The authors are committed to making professional choices acknowledging the climate emergency. We submitted this work to FoSSaCS for its excellence and because its location induces for us a low carbon footprint. This work was supported by the European Research Council (ERC) under the European Union's Horizon 2020 research and innovation programme (grant agreement No.670624), and by the DeLTA ANR project (ANR-16-CE40-0007).

Our main technical result is that the stochastic control problem is decidable. In the next paragraphs we discuss four motivations for studying this problem: control of biological systems, parameterised verification and control, distributed computing, and automata theory.

*Modelling biological systems.* The original motivation for studying this model was for controlling population of yeasts ([21]). In this application, the concentration of some molecule is monitored through fluorescence level. Controlling the frequency and duration of injections of a sorbitol solution influences the concentration of the target molecule, triggering different chemical reactions which can be modelled by a finite state system. The objective is to control the population to reach a predetermined fluorescence state. As discussed in the conclusions of [3,4], the stochastic semantics is more satisfactory than the adversarial one for representing the behaviours of the chemical reactions, so our decidability result is a step towards a better understanding of the modelling of biological systems as populations of arbitrarily many agents represented by finite state systems.

*From parameterised verification to parameterised control.* Parameterised verification was introduced in [12]: it is the verification of a system composed of an arbitrary number of identical components. The control problem we study here and introduced in [3,4] is the first step towards *parameterised control*: the goal is control a system composed of many identical components in order to ensure a given property. To the best of our knowledge, the contributions of [3,4] are the first results on parameterised control; by extension, we present the first results on parameterised control in a stochastic setting.

*Distributed computing.* Our model resembles two models introduced for the study of distributed computing. The first and most widely studied is population protocols, introduced in [2]: the agents are modelled by finite state systems and interact by pairs drawn at random. The mode of interaction is the key difference with the model we study here: in a time step, all of our agents perform simultaneously and independently the same action. This brings us closer to broadcast protocols as studied for instance in [8], in which one action involves an arbitrary number of agents. As explained in [3,4], our model can be seen as a subclass of (stochastic) broadcast protocols, but key differences exist in the semantics, making the two bodies of work technically independent.

The focus of the distributed computing community when studying population or broadcast protocols is to construct the most efficient protocols for a given task, such as (prominently) electing a leader. A growing literature from the verification community focusses on checking the correctness of a given protocol against a given specification; we refer to the recent survey [7] for an overview. We concentrate on the control problem, which can then be seen as a first result in the control of distributed systems in a stochastic setting.

*Alternative semantics for probabilistic automata.* It is very tempting to consider the limit case of infinitely many agents: the parameterised control question becomes the value 1 problem for probabilistic automata, which was proved undecidable in [13], and even in very restricted cases ([10]). Hence abstracting continuous distributions by a discrete population of arbitrary size can be seen as an approximation technique for probabilistic automata. Using $n$ agents correponds to using numerical approximation up to $2^{-n}$ with random rounding; in this sense the control problem considers arbitrarily fine approximations. The plague of undecidability results on probabilistic automata (see *e.g.* [9]) is nicely contrasted by our positive result, which is one of the few decidability results on probabilistic automata not making structural assumptions on the underlying graph.

*Our results.* We prove decidability of the stochastic control problem. The first insight is given by the theory of well quasi orders, which motivates the introduction of a new problem called the sequential flow problem. The first step of our solution is to reduce the stochastic control problem to (many instances of) the sequential flow problem. The second insight comes from the theory of regular cost functions, providing us with a set of tools for addressing the key difficulty of the problem, namely the fact that there are arbitarily many agents. Our key technical contribution is to show the computability of the sequential flow problem by reducing it to a boundedness question expressed in the cost monadic second order logic using the max-flow min-cut theorem.

*Related work.* The notion of decisive Markov chains was introduced in [1] as a unifying property for studying infinite-state Markov chains with finite-like properties. A typical example of decisive Markov chains is lossy channel systems where tokens can be lost anytime inducing monotonicity properties. Our situation is the exact opposite as we are considering (using the Petri nets terminology) safe Petri nets where the number of tokens along a run is constant. So it is not clear whether the underlying argument in both cases can be unified using decisiveness.

*Organisation of the paper.* We define the stochastic control problem in Section 2, and the sequential flow problem in Section 3. We construct a reduction from the former to (many instances of) the latter in Section 4, and show the decidability of the sequential flow problem in Section 5.

## 2 The stochastic control problem

**Definition 1.** *A* Markov decision process *(*MDP *for short) consists of*

- *a finite set of* states $\mathcal{Q}$*,*
- *a finite set of* actions $\mathcal{A}$*,*
- *a stochastic* transition table $\rho : \mathcal{Q} \times \mathcal{A} \to \mathcal{D}(\mathcal{Q})$*.*

The interpretation of the transition table is that from the state $p$ under action $a$, the probability to transition to $q$ is $\rho(p, a)(q)$. The *transition relation* $\Delta$ is defined by

$$\Delta = \{(p, a, q) \in \mathcal{Q} \times \mathcal{A} \times \mathcal{Q} : \rho(p, a)(q) > 0\}.$$

We also use $\Delta_a$ given by $\{(p, q) \in Q \times Q : (p, a, q) \in \Delta\}$.

We refer to [17] for the usual notions related to MDPs; it turns out that very little probability theory will be needed in this paper, so we restrict ourselves to mentioning only the relevant objects. In an MDP $\mathcal{M}$, a strategy is a function $\sigma : \mathcal{Q} \to \mathcal{A}$; note that we consider only pure and positional strategies, as they will be sufficient for our purposes.

Given a *source* $s \in \mathcal{Q}$ and a *target* $t \in \mathcal{Q}$, we say that the strategy $\sigma$ *almost surely* reaches $t$ if the probability that a path starting from $s$ and consistent with $\sigma$ eventually leads to $t$ is 1. As we shall recall in Section 4, whether there exists a strategy ensuring to reach $t$ almost surely from $s$, called the *almost sure reachability problem* for MDP can be reduced to solving a two player Büchi game, and in particular does not depend upon the exact probabilities. In other words, the only relevant information for each $(p, a, q) \in \mathcal{Q} \times \mathcal{A} \times \mathcal{Q}$ is whether $\rho(p, a)(q) > 0$ or not. Since the same will be true for the stochastic control problem we study in this paper, in our examples we do not specify the exact probabilities, and an edge from $p$ to $q$ labelled $a$ means that $\rho(p, a)(q) > 0$.

Let us now fix an MDP $\mathcal{M}$ and consider a population of $n$ *tokens* (we use tokens to represent the agents). Each token evolves in an independent copy of the MDP $\mathcal{M}$. The controller acts through a *strategy* $\sigma : \mathcal{Q}^n \to \mathcal{A}$, meaning that given the state each of the $n$ tokens is in, the controller chooses *one* action to be performed by all tokens independently. Formally, we are considering the product MDP $\mathcal{M}^n$ whose set of states is $\mathcal{Q}^n$, set of actions is $\mathcal{A}$, and transition table is $\rho^n(u, a)(v) = \prod_{i=1}^{n} \rho(u_i, a)(v_i)$, where $u, v \in \mathcal{Q}^n$ and $u_i, v_i$ are the $i^{\text{th}}$ components of $u$ and $v$.

Let $s, t \in \mathcal{Q}$ be the source and target states, we write $s^n$ and $t^n$ for the constant $n$-tuples where all components are $s$ and $t$. For a fixed value of $n$, whether there exists a strategy ensuring to reach $t^n$ almost surely from $s^n$ can be reduced to solving a two player Büchi game in the same way as above for a single MDP, replacing $\mathcal{M}$ by $\mathcal{M}^n$. The stochastic control problem asks whether this is true for arbitrary values of $n$:

*Problem 1 (*Stochastic control problem*).* The inputs are an MDP $\mathcal{M}$, a source state $s \in \mathcal{Q}$ and a target state $t \in \mathcal{Q}$. The question is whether for all $n \in \mathbb{N}$, there exists a strategy ensuring to reach $t^n$ almost surely from $s^n$.

Our main result is the following.

**Theorem 1.** *The stochastic control problem is decidable.*

The fact that the problem is co-recursively enumerable is easy to see: if the answer is "no", there exists $n \in \mathbb{N}$ such that there exist no strategy ensuring to reach $t^n$ almost surely from $s^n$. Enumerating the values of $n$ and solving the almost sure reachability problem for $\mathcal{M}^n$ eventually finds this out. However, it is not clear whether one can place an upper bound on such a witness $n$, which would yield a simple (yet inefficient!) algorithm. As a corollary of our analysis we can indeed derive such an upper bound, but it is non elementary in the size of the MDP.

In the remainder of this section we present a few interesting examples.

**Example 1** Let us consider the MDP represented in Figure 1. We show that for this MDP, for any $n \in \mathbb{N}$, the controller has an almost sure strategy to reach $t^n$ from $s^n$. Starting with $n$ tokens on $s$, we iterate the following strategy:

– Repeatedly play action $a$ until all tokens are in $q$;
– Play action $b$.

The first step is eventually successful with probability one, since at each iteration there is a positive probability that the number of tokens in state $q$ increases. In the second step, with non zero probability at least one token goes to $t$, while the rest go back to $s$. It follows that each iteration of this strategy increases with non zero probability the number of tokens in $t$. Hence, all tokens are eventually transferred to $t^n$ almost surely.

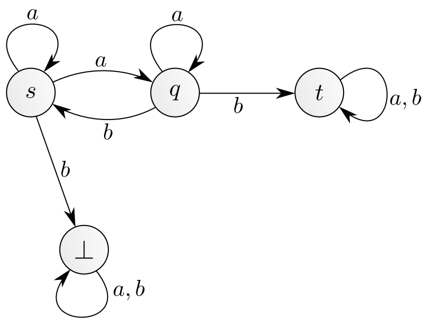

**Fig. 1.** The controller can almost surely reach $t^n$ from $s^n$, for any $n \in \mathbb{N}$.

**Example 2** We now consider the MDP represented in Figure 2. By convention, if from a state some action does not have any outgoing transition (for instance the action $u$ from $s$), then it goes to the sink state $\bot$.

We show that there exists a controller ensuring to transfer seven tokens from $s$ to $t$, but that the same does not hold for eight tokens. For the first assertion, we present the following strategy:

– Play $a$. One of the states $q_1^{i_1}$ for $i_1 \in \{u, d\}$ receives at least 4 tokens.
– Play $i_1 \in \{u, d\}$. At least 4 tokens go to $t$ while at most 3 go to $q_1$.
– Play $a$. One of the states $q_2^{i_2}$ for $i_2 \in \{u, d\}$ receives at least 2 tokens.
– Play $i_2 \in \{u, d\}$. At least 2 tokens go to $t$ while at most 1 token goes to $q_2$.
– Play $a$. The token (if any) goes to $q_3^i$ for $i_3 \in \{u, d\}$.

– Play $i_3 \in \{u, d\}$. The remaining token (if any) goes to $t$.

Now assume that there are 8 tokens or more on $s$. The only choices for a strategy are to play $u$ or $d$ on the second, fourth, and sixth move. First, with non zero probability at least 4 tokens are in each of $q_1^i$ for $i \in \{u, d\}$. Then, whatever the choice of action $i \in \{u, d\}$, there are at least 4 tokens in $q_1$ after the next step. Proceeding likewise, there are at least 2 tokens in $q_2$ with non zero probability two steps later. Then again two steps later, at least 1 token falls in the sink with non zero probability.

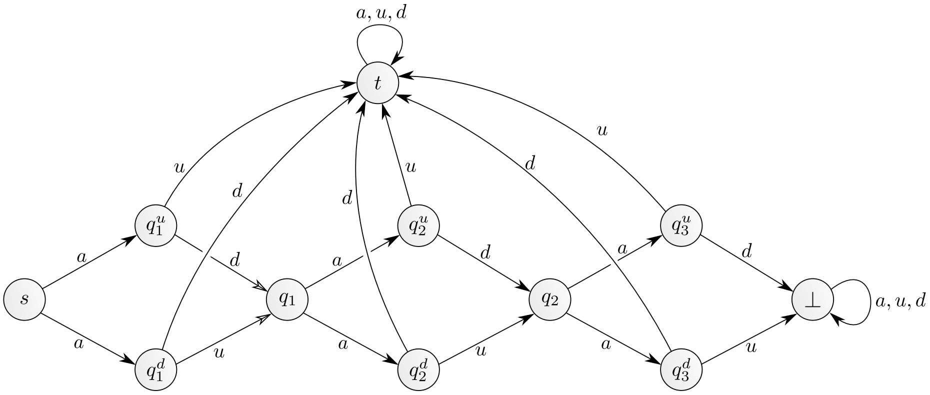

**Fig. 2.** The controller can synchronise up to 7 tokens on the target state $t$ almost surely, but not more.

Generalising this example shows that if the answer to the stochastic control problem is “no”, the smallest number of tokens $n$ for which there exist no almost surely strategy for reaching $t^n$ from $s^n$ may be exponential in $|\mathcal{Q}|$. This can further extended to show a doubly exponential in $\mathcal{Q}$ lower bound, as done in [3,4]; the example produced there holds for both the adversarial and the stochastic setting. Interestingly, for the adversarial setting this doubly exponential lower bound is tight. Our proof for the stochastic setting yields a non-elementary bound, leaving a very large gap.

**Example 3** We consider the MDP represented in Figure 3. For any $n \in \mathbb{N}$, there exists a strategy almost surely reaching $t^n$ from $s^n$. However, this strategy has to pass tokens one by one through $q_1$. We iterate the following strategy:

– Repeatedly play action $a$ until exactly 1 token is in $q_1$.
– Play action $b$. The token goes to $q_i$ for some $i \in \{l, r\}$.
– Play action $i \in \{l, r\}$, which moves the token to $t$.

Note that the first step may take a very long time (the expectation of the number of $a$s to be played until this happens is exponential in the number of tokens), but it is eventually successful with probability one. This very slow strategy is necessary: if $q_1$ contains at least two tokens, then action $b$ should not be played: with non zero probability, at least one token ends up in each of $q_l, q_r$, so at the next step some token ends up in $\bot$. It follows that any strategy almost surely reaching $t^n$ has to be able to detect the presence of at most 1 token in $q_1$. This is a key example for understanding the difficulty of the stochastic control problem.

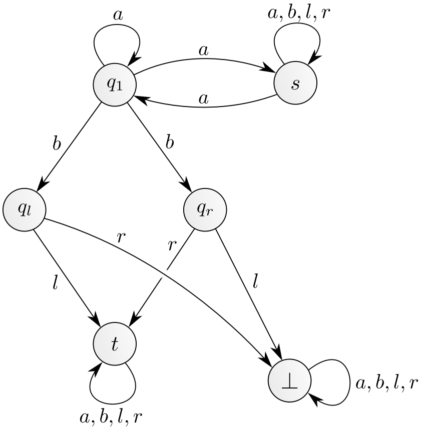

**Fig. 3.** The controller can synchronise any number of tokens almost surely on the target state $t$, but they have to go one by one.

## 3 The sequential flow problem

We let $Q$ be a finite set of states. We call *configuration* an element of $\mathbb{N}^Q$ and *flow* an element of $f \in \mathbb{N}^{Q \times Q}$. A flow $f$ induces two configurations $\mathrm{pre}(f)$ and $\mathrm{post}(f)$ defined by

$$\mathrm{pre}(f)(p) = \sum_{q \in Q} f(p,q) \qquad \text{and} \qquad \mathrm{post}(f)(q) = \sum_{p \in Q} f(p,q).$$

Given $c, c'$ two configurations and $f$ a flow, we say that $c$ *goes to* $c'$ using $f$ and write $c \to^f c'$, if $c = \mathrm{pre}(f)$ and $c' = \mathrm{post}(f)$.

A *flow word* is $f = f_1 \dots f_\ell$ where each $f_i$ is a flow. We write $c \rightsquigarrow^f c'$ if there exists a sequence of configurations $c = c_0, c_1, \dots, c_\ell = c'$ such that $c_{i-1} \to^{f_i} c_i$ for all $i \in \{1, \dots, \ell\}$. In this case, we say that $c$ goes to $c'$ using the flow word $f$.

We now recall some classical definitions related to well quasi orders ([15,16], see [19] for an exposition of recent results). Let $(E, \leqslant)$ be a quasi ordered set (*i.e.* $\leqslant$ is reflexive and transitive), it is a *well quasi ordered set* (WQO) if any infinite sequence contains an increasing pair. We say that $S \subseteq E$ is *downward closed* if for any $x \in S$, if $y \leqslant x$ then $y \in S$. An *ideal* is a non-empty downward closed set $I \subseteq E$ such that for all $x, y \in I$, there exists some $z \in I$ satisfying both $x \leqslant z$ and $y \leqslant z$.

**Lemma 1.**

- *Any infinite sequence of decreasing downward closed sets in a WQO is eventually constant.*
- *A subset is downward closed if and only if it is a finite union of incomparable ideals. We call it its* decomposition into ideals *(or simply, its* decomposition*), which is unique (up to permutation).*
- *An ideal is included in a downward closed set if and only if it is included in one of the ideals of its decomposition.*

We equip the set of configurations $\mathbb{N}^{\mathcal{Q}}$ and the set of flows $\mathbb{N}^{\mathcal{Q}\times\mathcal{Q}}$ with the quasi order $\leqslant$ defined component wise, yielding thanks to Dickson's Lemma [6] two WQOs.

**Lemma 2.** *Let $X$ be a finite set. A subset of $\mathbb{N}^X$ is an ideal if and only if it is of the form*

$$a{\downarrow} = \{c \in \mathbb{N}^X \mid c \leqslant a\},$$

*for some $a \in (\mathbb{N} \cup \{\omega\})^X$ (in which $\omega$ is larger than all integers).*

We represent downward closed sets of configurations and flows using their decomposition into finitely many ideals of the form $a{\downarrow}$ for $a \in (\mathbb{N} \cup \{\omega\})^{\mathcal{Q}}$ or $a \in (\mathbb{N} \cup \{\omega\})^{\mathcal{Q}\times\mathcal{Q}}$.

*Problem 2 (*Sequential flow problem*).* Let $\mathcal{Q}$ be a finite set of states. Given a downward closed set of flows $\mathit{Flows} \subseteq \mathbb{N}^{\mathcal{Q}\times\mathcal{Q}}$ and a downward closed set of final configurations $F \subseteq \mathbb{N}^{\mathcal{Q}}$, compute the downward closed set

$$\mathrm{Pre}^*(\mathit{Flows}, F) = \{c \in \mathbb{N}^{\mathcal{Q}} \mid c \leadsto^{f} c' \in F,\ f \in \mathit{Flows}^*\}\ ,$$

i.e. the configurations from which one may reach $F$ using only flows from *Flows*.

## 4 Reduction of the stochastic control problem to the sequential flow problem

Let us consider an MDP $\mathcal{M}$ and a target $t \in \mathcal{Q}$. We first recall a folklore result reducing the almost sure reachability question for MDPs to solving a two player Büchi game (we refer to [14] for the definitions and notations of Büchi games). The Büchi game is played between *Eve* and *Adam* as follows. From a state $p$:

1. Eve chooses an action $a$ and a transition $(p, q) \in \Delta_a$;
2. Adam can either choose to
   **agree** and the game continues from $q$, or
   **interrupt** and choose another transition $(p, q') \in \Delta_a$, the game continues from $q'$.

The Büchi objective is satisfied (meaning Eve wins) if either the target state $t$ is reached or Adam interrupts infinitely many times.

**Lemma 3.** *There exists a strategy ensuring almost surely to reach $t$ from $s$ if and only if Eve has a winning strategy from $s$ in the above Büchi game.*

We now explain how this reduction can be extended to the stochastic control problem. Let us consider an MDP $\mathcal{M}$ and a target $t \in \mathcal{Q}$. We now define an infinite Büchi game $\mathcal{G}_{\mathcal{M}}$. The set of vertices is the set of configurations $\mathbb{N}^{\mathcal{Q}}$. For a flow $f$, we write $\mathrm{supp}(f) = \{(p,q) \in \mathcal{Q}^2 : f(p,q) > 0\}$. The game is played as follows from a configuration $c$:

1. Eve chooses an action $a$ and a flow $f$ such that $\mathrm{pre}(f) = c$ and $\mathrm{supp}(f) \subseteq \Delta_a$.
2. Adam can either choose to

***agree*** and the game continues from $c' = \mathrm{post}(f)$

***interrupt*** and choose a flow $f'$ such that $\mathrm{pre}(f') = c$ and $\mathrm{supp}(f') \subseteq \Delta_a$, and the game continues from $c'' = \mathrm{post}(f')$.

Note that Eve choosing a flow $f$ is equivalent to choosing for each token a transition $(p,q) \in \Delta_a$, inducing the configuration $c'$, and simiarly for Adam should he decide to interrupt.

Eve wins if either all tokens are in the target state, or if Adam interrupts infinitely many times.

Note that although the game is infinite, it is actually a disjoint union of finite games. Indeed, along a play the number of tokens is fixed, so each play is included in $\mathcal{Q}^n$ for some $n \in \mathbb{N}$.

**Lemma 4.** *Let $c$ be a configuration with $n$ tokens in total, the following are equivalent:*

– *There exists a strategy almost surely reaching $t^n$ from $c$,*
– *Eve has a winning strategy in the Büchi game $\mathcal{G}_{\mathcal{M}}$ starting from $c$.*

Lemma 4 follows from applying Lemma 3 on the product MDP $\mathcal{M}^n$.

We also consider the game $\mathcal{G}_{\mathcal{M}}^{(i)}$ for $i \in \mathbb{N}$, which is defined just as $\mathcal{G}_{\mathcal{M}}$ except for the winning objective: Eve wins in $\mathcal{G}_{\mathcal{M}}^{(i)}$ if either all tokens are in the target state, or if Adam interrupts more than $i$ times. It is clear that if Eve has a winning strategy in $\mathcal{G}_{\mathcal{M}}$ then she has a winning strategy in $\mathcal{G}_{\mathcal{M}}^{(i)}$. Conversely, the following result states that $\mathcal{G}_{\mathcal{M}}^{(i)}$ is equivalent to $\mathcal{G}_{\mathcal{M}}$ for some $i$.

**Lemma 5.** *There exists $i \in \mathbb{N}$ such that from any configuration $c \in \mathbb{N}^{\mathcal{Q}}$, Eve has a winning strategy in $\mathcal{G}_{\mathcal{M}}$ if and only if Eve has a winning strategy in $\mathcal{G}_{\mathcal{M}}^{(i)}$.*

*Proof:* Let $X^{(i)} \subseteq \mathbb{N}^{\mathcal{Q}}$ be the winning region for Eve in $\mathcal{G}_{\mathcal{M}}^{(i)}$. We first argue that $X = \bigcap_i X^{(i)}$ is the winning region in $\mathcal{G}_{\mathcal{M}}$. It is clear that $X$ is contained in the winning region: if Eve has a strategy to ensure that either all tokens are in the target state, or that Adam interrupts infinitely many times, then it particular this is true for Adam interrupting more than $i$ times for any $i$. The converse inclusion holds because $\mathcal{G}_{\mathcal{M}}$ is a disjoint union of finite Büchi games. Indeed, in a finite Büchi game, since Adam can restrict himself to playing a memoryless winning strategy, if Eve can ensure that he interrupts a certain number of times (larger than the size of the game), then by a simple pumping argument this implies that Adam will interrupt infinitely many times.

To conclude, we note that each $X^{(i)}$ is downward closed: indeed, a winning strategy from a configuration $c$ can be used from a configuration $c'$ where there are fewer tokens in each state. It follows that $(X^{(i)})_{i \geq 0}$ is a decreasing sequence of downward closed sets in $\mathbb{N}^{\mathcal{Q}}$, hence it stabilises thanks to Lemma 1, *i.e.* there exists $i_0 \in \mathbb{N}$ such that $X^{(i_0)} = \bigcap_i X^{(i)}$, which concludes. □

Note that Lemma 4 and Lemma 5 substantiate the claims made in Section 2: pure positional strategies are enough and the answer to the stochastic control problem does not depend upon the exact probabilities in the MDP. Indeed, the construction of the Büchi games do not depend on them, and the answer to the former is equivalent to determining whether Eve has a winning strategy in each of them.

We are now fully equipped to show that a solution to the sequential flow problem yields the decidability of the stochastic control problem.

Let $F$ be the set of configurations for which all tokens are in state $t$. we let $X^{(i)} \subseteq \mathbb{N}^{\mathcal{Q}}$ denote the winning region for Eve in the game $\mathcal{G}_{\mathcal{M}}^{(i)}$. Note first that $X^{(0)} = \mathrm{Pre}^*(\mathit{Flows}^0, F)$ where

$$\mathit{Flows}^0 = \{f \in \mathbb{N}^{\mathcal{Q} \times \mathcal{Q}} : \exists a \in \mathcal{A},\ \mathrm{supp}(f) \subseteq \Delta_a\}.$$

Indeed, in the game $\mathcal{G}_{\mathcal{M}}^{(0)}$ Adam cannot interrupt as this would make him lose immediately. Hence, the winning region for Eve in $\mathcal{G}_{\mathcal{M}}^{(0)}$ is $\mathrm{Pre}^*(\mathit{Flows}^0, F)$.

We generalise this by setting $\mathit{Flows}^i$ for all $i > 0$ to be the set of flows $f \in \mathbb{N}^{\mathcal{Q} \times \mathcal{Q}}$ such that for some action $a \in \mathcal{A}$,

- $\mathrm{supp}(f) \subseteq \Delta_a$, and
- for $f'$ with $\mathrm{pre}(f') = \mathrm{pre}(f)$ and $\mathrm{supp}(f') \subseteq \Delta_a$, we have $\mathrm{post}(f') \in X^{(i-1)}$.

Equivalently, this is the set of flows for which, when played in the game $\mathcal{G}_{\mathcal{M}}$ by Eve, Adam cannot use an interrupt move and force the configuration outside of $X^{(i-1)}$.

We now claim that

$$X^{(i)} = \mathrm{Pre}^*(\mathit{Flows}^i, F)$$

for all $i \geq 0$.

We note that this means that for each $i$ computing $X^{(i)}$ reduces to solving one instance of the sequential flow problem. This induces an algorithm for solving the stochastic control problem: compute the sequence $(X^{(i)})_{i\geq 0}$ until it stabilises, which is ensured by Lemma 5 and yields the winning region of $\mathcal{G}_{\mathcal{M}}$. The answer to the stochastic control problem is then whether the initial configuration where all tokens are in $s$ belongs to the winning region of $\mathcal{G}_{\mathcal{M}}$.

Let us prove the claim by induction on $i$.

Let $c$ be a configuration in $\mathrm{Pre}^*(\mathit{Flows}^i, F)$. This means that there exists a flow word $f = f_1 \cdots f_\ell$ such that $f_k \in \mathit{Flows}^i$ for all $k$, and $c \rightsquigarrow^f c' \in F$. Expanding the definition, there exist $c_0 = c, \dots, c_\ell = c'$ such that $c_{k-1} \rightarrow^{f_k} c_k$ for all $k$.

Let us now describe a strategy for Eve in $\mathcal{G}_{\mathcal{M}}^{(i)}$ starting from $c$. As long as Adam agrees, Eve successively chooses the sequence of flows $f_1, f_2, \dots$ and the corresponding configurations $c_1, c_2, \dots$. If Adam never interrupts, then the game reaches the configuration $c' \in F$, and Eve wins. Otherwise, as soon as Adam interrupts, by definition of $\mathit{Flows}^i$, we reach a configuration $d \in X^{(i-1)}$. By induction hypothesis, Eve has a strategy which ensures from $d$ to either reach $F$ or that Adam interrupts at least $i-1$ times. In the latter case, adding the interrupt move leading to $d$ yields $i$ interrupts, so this is a winning strategy for Eve in $\mathcal{G}_{\mathcal{M}}^{(i)}$, witnessing that $c \in X^{(i)}$.

Conversely, assume that there is a winning strategy $\sigma$ of Eve in $\mathcal{G}_{\mathcal{M}}^{(i)}$ from a configuration $c$. Consider a play consistent with $\sigma$, it either reaches $F$ or Adam interrupts. Let us denote by $f = f_1, f_2, \dots, f_\ell$ the sequence of flows until then. We argue that $f_k \in \mathit{Flows}^i$ for $k \in \{1, \dots, \ell\}$. Let $f = f_k$ for some $k$, by definition of the game $\mathrm{supp}(f) \subseteq \Delta_a$ for some action $a$. Let $f'$ such that $\mathrm{pre}(f') = \mathrm{pre}(f)$ and $\mathrm{supp}(f') \subseteq \Delta_a$. In the game $\mathcal{G}_{\mathcal{M}}$ after Eve played $f_k$, Adam has the possibility to interrupt and choose $f'$. From this configuration onward the strategy $\sigma$ is winning in $\mathcal{G}_{\mathcal{M}}^{(i-1)}$, implying that $f \in \mathit{Flows}^i$. Thus $f = f_1 f_2 \dots f_\ell$ is a witness that $c \in X^{(i)}$.

## 5 Computability of the sequential flow problem

Let $Q$ be a finite set of states, $\mathit{Flows} \subseteq \mathbb{N}^{Q\times Q}$ a downward closed set of flows and $F \subseteq \mathbb{N}^Q$ a downward closed set of configurations, the sequential flow problem is to compute the downward closed set $\mathrm{Pre}^*$ defined by

$$\mathrm{Pre}^*(\mathit{Flows}, F) = \{c \in \mathbb{N}^Q \mid c \rightsquigarrow^f c' \in F,\ f \in \mathit{Flows}^*\} \ ,$$

*i.e.* the configurations from which one may reach $F$ using only flows from $\mathit{Flows}$.

The following classical result of [22] allows us to further reduce our problem.

**Lemma 6.** *The task of computing a downward closed set can be reduced to the task of deciding whether a given ideal is included in a downward closed set.*

Thanks to Lemma 6, it is sufficient for solving the sequential flow problem to establish the following result.

**Lemma 7.** *Let $I$ be an ideal of the form $a\downarrow$ for $a \in (\mathbb{N} \cup \{\omega\})^{\mathcal{Q}}$, and Flows $\subseteq \mathbb{N}^{\mathcal{Q}\times\mathcal{Q}}$ be a downward closed set of flows. It is decidable whether $F$ can be reached from all configurations of $I$ using only flows from Flows.*

We call a vector $a \in (\mathbb{N} \cup \{\omega\})^{\mathcal{Q}\times\mathcal{Q}}$ a *capacity.* A *capacity word* is a finite sequence of capacities. For two capacity words $w, w'$ of the same length, we write $w \leq w'$ to mean that $w_i \leq w'_i$ for each $i$. Since flows are particular cases of capacities, we can compare flows with capacities in the same way.

Before proving Lemma 7 let us give an example and some notations.

Given a state $q$, we write $q \in \mathbb{N}^{\mathcal{Q}}$ for the vector which has value 1 on the $q$ component and 0 elsewhere. More generally we let $\alpha q$ for $\alpha \in \mathbb{N} \cup \{\omega\}$ denote the vector with value $\alpha$ on the $q$ component and 0 elsewhere. We use similar notations for flows. For instance, $\omega q_1 + q_2$ has value $\omega$ in the $q_1$ component, 1 in the $q_2$ component, and 0 elsewhere.

In the instance of the sequential flow problem represented in Figure 4, we ask the following question: can $F$ be reached from any configuration of $I = (\omega q_2)\downarrow$? The answer is yes: the capacity word $w = (ac^{n-1}b)^n$ is such that $nq_2 \rightsquigarrow^f nq_4 \in F$ for a flow word $f \leqslant w$, the begining of which is described in Figure 5.

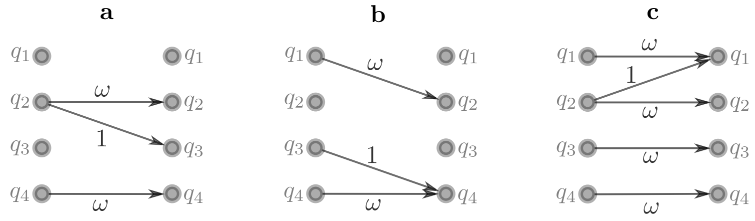

**Fig. 4.** An instance of the sequential flow problem. We let *Flows* $= a\downarrow \cup\, b\downarrow \cup\, c\downarrow$ where $a = \omega(q_2, q_2) + (q_2, q_3) + \omega(q_4, q_4)$, $b = \omega(q_1, q_2) + (q_3, q_4) + \omega(q_4, q_4)$, and $c = \omega(q_1, q_1) + (q_2, q_1) + \omega(q_2, q_2) + \omega(q_3, q_3) + \omega(q_4, q_4)$. Set also $F = (\omega q_4)\downarrow$.

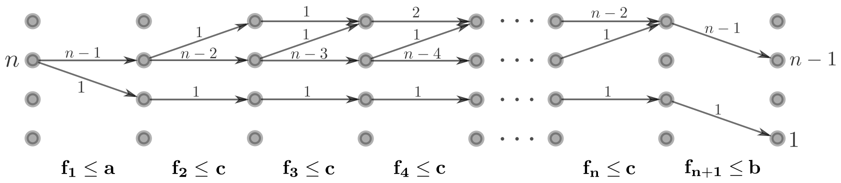

**Fig. 5.** A flow word $f = f_1 f_2 \ldots f_{n+1} \leqslant ac^{n-1}b$ such that $nq_2$ goes to $(n-1)q_1 + q_4$ using $f$. This construction can be extended to $f \leqslant w$ such that $nq_2$ goes to $nq_4$ using $f$.

We write $a[\omega \leftarrow n]$ for the configuration obtained from $a$ by replacing all $\omega$s by $n$.

The key idea for solving the sequential flow problem is to rephrase it using *regular cost functions* (a set of tools for solving boundedness questions). Indeed, whether $F$ can be reached from all configurations of $I = a\downarrow$ using only flows from *Flows* can be equivalently phrased as a boundedness question, as follows:

> does there exist a bound on the values of $n \in \mathbb{N}$ such that $a[\omega \leftarrow n] \rightsquigarrow^{f} c$ for some $c \in F$ and $f \in \mathit{Flows}^*$?

We show that this boundedness question can be formulated as a boundedness question for a formula of *cost monadic logic*, a formalism that we introduce now. We assume that the reader is familiar with *monadic second order logic* (MSO) over finite words, and refer to [20] for the definitions. The syntax of cost monadic logic (cost MSO for short) extends MSO with the construct $|X| \leq N$, where $X$ is a second order variable and $N$ is a bounding variable. The semantics is defined as usual: $w, n \models \varphi$ for a word $w \in A^*$, with $n \in \mathbb{N}$ specifying the bound $N$. We assume that there is at most one bounding variable, and that the construct $|X| \leq N$ appears positively, *i.e.* under an even number of negations. This ensures that the larger $N$, the more true the formula is: if $w, n \models \varphi$, then $w, n' \models \varphi$ for all $n' \geq n$. The semantics of a formula $\varphi$ of cost MSO induces a function $A^* \to \mathbb{N} \cup \{\infty\}$ defined by $\varphi(w) = \inf \{n \in \mathbb{N} \mid w, n \models \varphi\}$.

The *boundedness problem* for cost monadic logic is the following problem: given a cost MSO formula $\varphi$ over $A^*$, is it true that the function $A^* \to \mathbb{N} \cup \{\infty\}$ is bounded, *i.e.*:

$$\exists n \in \mathbb{N},\ \forall w \in A^*,\ w, n \models \varphi?$$

The decidability of the boundedness problem is a central result in the theory of regular cost functions ([5]). Since in the theory of regular cost functions, when considering functions we are only interested in whether they are bounded or not, we will consider functions "up to boundedness properties". Concretely, this means that a *cost function* is an equivalence class of functions $A^* \to \mathbb{N} \cup \{\infty\}$, with the equivalence being $f \approx g$ if there exists $\alpha : \mathbb{N} \to \mathbb{N}$ such that $f(w)$ is finite if and only if $g(w)$ is finite, and in this case, $f(w) \leqslant \alpha(g(w))$ and $g(w) \leqslant \alpha(f(w))$. This is equivalent to stating that for all $X \subseteq A^*$, if $f$ is bounded over $X$ if and only if $g$ is bounded over $X$.

Let us now establish Lemma 7.

*Proof:* Let $T = \{q \in \mathcal{Q} \mid a(q) = \omega\}$. Note that for $n$ sufficiently large, we have $a[\omega \leftarrow n]\downarrow = I \cap \{0, 1, \ldots, n\}$. We let $\mathscr{C} \subseteq (\mathbb{N} \cup \{\omega\})^{\mathcal{Q} \times \mathcal{Q}}$ be the decomposition of *Flows* into ideals, that is, $\mathscr{C}$ is the minimal finite set such that

$$\mathit{Flows} = \bigcup_{b \in \mathscr{C}} b\downarrow .$$

We let $k$ denote the largest finite value that appears in the definition of $\mathscr{C}$, that is, $k = \max\{b(q, q') : b \in \mathscr{C}, q, q' \in \mathcal{Q}, b(q, q') \neq \omega\}$.

Let us define the function

$$\begin{aligned} \Phi : \mathscr{C}^* &\longrightarrow \mathbb{N} \cup \{\omega\} \\ w &\longmapsto \sup\{n \in \mathbb{N} : \exists f \leqslant w, a[\omega \leftarrow n] \rightsquigarrow^{f} F\}. \end{aligned}$$

By definition $\Phi$ is unbounded if and only if $F$ can be reached from all configurations of $I$. Since boundedness of cost MSO is decidable, it suffices to construct a formula in cost monadic logic for $\Phi$ to obtain the decidability of our problem. Our approach will be to additively decompose the capacity word $w$ into a finitary part $w^{(\text{fin})}$ (which is handled using a regular language), and several unbounded parts $w^{(s)}$ for each $s \in T$. The unbounded parts require a more careful analysis which notably goes through the use of the max-flow min-cut theorem.

Note that $a[\omega \leftarrow n]$ decomposes as the sum of its finite part $a_{\text{fin}} = a[\omega \leftarrow 0]$ and $\sum_{s\in T} ns$. Since flows are additive, it holds that $f \leqslant w = w_1 \dots w_l$ is a flow from $c_n$ to $F$ if and only if the capacity word $w$ may be decomposed into $(w^{(s)})_{s\in T} = (w_1^{(s)} \dots w_l^{(s)})_{s\in T}$ and $w^{(\text{fin})} = w_1^{(\text{fin})} \dots w_l^{(\text{fin})}$ such that

– all the numbers appearing in the $w_i^{(s)}$ capacities are bounded by $k$,
– for all $i \in \{1, \dots, l\}, w_i = \sum_{s\in T\cup\{fin\}} w_i^{(s)}$,
– for all $s \in T$, $ns \rightsquigarrow^f F$ for some flow word $f \leqslant w^{(s)}$,
– and $a_{\text{fin}} \rightsquigarrow^f F$ for some flow word $f \leqslant w^{(\text{fin})}$.

In order to encode such capacity words in cost MSO we use monadic variables $W^{(s)}_{q,q',p}$ where $q, q' \in \mathcal{Q}$, $p \in \{0, \dots, k, \omega\}$ and $s \in T \cup \{\text{fin}\}$. They are meant to satisfy that $i \in W^{(s)}_{q,q',p,s}$ if and only if $w_i^{(s)}(q, q') = p$. We use bold $\boldsymbol{W}$ to denote the tuple $(W^{(s)}_{q,q',p})_{q,q',p,s}$, and $\boldsymbol{W}^{(s)}$ for $(W^{(s)}_{q,q',p})_{q,q',p}$ when $s \in T \cup \{\omega\}$ is fixed. The MSO formula $\texttt{IsDecomp}(\boldsymbol{W}, w)$ states that a decomposition $(w^{(s)})_{s\in T\cup\{\omega\}}$ is semantically valid and sums to $w$:

$$\begin{aligned} \forall i, \quad & \Big[\bigwedge\nolimits_{q,q',s} \bigvee\nolimits_{p\in\{0,\dots,k,\omega\}} \Big(i \in W^{(s)}_{q,q',p} \wedge \bigwedge\nolimits_{p'\neq p} i \notin W^{(s)}_{q,q',p}\Big)\Big] \\ \wedge & \left[\Big(\bigwedge\nolimits_{q,q'p} w_i(q, q') = p\Big) \implies \bigvee\nolimits_{\substack{(p_s)_{s\in T\cup\{\text{fin}\}} \\ \sum p_s = p}} \bigwedge\nolimits_{s\in T\cup\{\text{fin}\}} i \in W^{(s)}_{q,q',p_s}\right] \end{aligned}$$

For $s \in T$, we now consider the function

$$\begin{aligned} \Psi^{(s)} : \big(\{0, 1, \dots, k, \omega\}^{\mathcal{Q}\times\mathcal{Q}}\big)^* &\longrightarrow \mathbb{N} \cup \{\omega\} \\ w^{(s)} &\longmapsto \sup\{n \in \mathbb{N} \mid \exists f \leqslant w^{(s)},\ ns \overset{f}{\rightsquigarrow} F\}. \end{aligned}$$

We also define $\Psi^{(\text{fin})} \subseteq (\{0, \dots, k, \omega\})^{\mathcal{Q}\times\mathcal{Q}}$ to be the language of capacity words $w^{(\text{fin})}$ such that there exists a flow $f \leqslant w^{(\text{fin})}$ with $a_{\text{fin}} \rightsquigarrow^f F$. Note that $\Psi^{(\text{fin})}$ is a regular language since it is recognized by a finite automaton over $\{0, 1, \dots, k|Q|\}^{\mathcal{Q}}$ that may update the current bounded configuration only with flows smaller than the current letter of $w^{(\text{fin})}$.

We have

$$\Phi(w) = \sup_n \Big[\exists \boldsymbol{W}, \texttt{IsDecomp}(\boldsymbol{W}, w) \wedge \Big(\bigwedge_{s\in T} \Psi^{(s)}(\boldsymbol{W}^{(s)}) \geq n\Big) \wedge \boldsymbol{W}^{(\text{fin})} \in \Psi^{(\text{fin})}\Big].$$

Hence, it is sufficient to prove that for each $s \in T$, $\Psi^{(s)}$ is definable in cost MSO.

Let us fix $s$ and a capacity word $w \in \{0, \ldots, k, \omega\}^{\mathcal{Q}\times\mathcal{Q}}$ of length $|w| = \ell$. Consider the finite graph $G$ with vertex set $\mathcal{Q} \times \{0, 1, \ldots, \ell\}$ and for all $i \geq 1$, an edge from $(q, i-1)$ to $(q', i)$ labelled by $w_i(q, q')$. Then $\Psi^{(s)}(w)$ is the maximal flow from $(s, 0)$ to $(t, \ell)$ in $G$. We recall that a *cut* in a graph with distinguished source $s$ and target $t$ is a set of edges such that removing them disconnects $s$ and $t$. The *cost of a cut* is the sum of the weight of its edges. The *max-flow min-cut theorem* states that the maximal flow in a graph is exactly the minimal cost of a cut ([11]).

We now define a cost MSO formula $\tilde{\Psi}^{(s)}$ which is equivalent (in terms of cost functions) to the minimal cost of cut in the previous graph $G$ and thus to $\Psi^{(s)}$. In the following formula, $\boldsymbol{X} = (X_{q,q'})_{q,q'\in\mathcal{Q}}$ represents a cut in the graph: $i \in X_{q,q'}$ means that edge $((q, i-1), (q', i))$ belongs to the cut. Likewise, $\boldsymbol{P} = (P_{q,q'})_{q,q'\in\mathcal{Q}}$ represents paths in the graph. Let $\tilde{\Psi}^{(s)}(w)$ be defined by

$$\inf_n \left\{ \exists \boldsymbol{X} \Big[ \bigwedge_{q,q'} n \geq |X_{q,q'}| \Big] \wedge \Big( \forall i, i \in X_{q,q'} \implies w_i(q,q') < \omega \Big) \ \wedge \ \mathrm{Disc}_{s,t}(\boldsymbol{X}, w) \right\},$$

where $\mathrm{Disc}_{s,t}(\boldsymbol{X}, w)$ expresses that $\boldsymbol{X}$ disconnects $(s, 0)$ and $(t, \ell)$ in $G$. For instance $\mathrm{Disc}_{s,t}(\boldsymbol{X}, w)$ is defined by

$$\forall \boldsymbol{P}, \Bigg[ \Big( \forall i, \bigwedge_{q,q'} i \in P_{q,q'} \implies w_i(q,q') > 0 \Big) \wedge \Big( \bigvee_{q'} 0 \in P_{s,q'} \Big) \wedge \Big( \bigvee_{q} \ell \in P_{q,t} \Big) \wedge$$

$$\forall i \geq 1, \bigwedge_{q,q'} i \in P_{q,q'} \implies \Big( \bigvee_{q''} i-1 \in P_{q'',q} \Big) \Bigg] \implies \exists i, \bigvee_{q,q'} \big( i \in X_{q,q'} \wedge i \in P_{q,q'} \big).$$

Now $\tilde{\Psi}^{(s)}(w)$ does not exactly define the minimal total weight $\Phi^{(s)}(w)$ of a cut, but rather the minimal value over all cuts of the minimum over $(q, q') \in \mathcal{Q}^2$ of how many edges are of the form $((q, i-1), (q', i))$. This is good enough for our purposes since these two values are related by

$$\tilde{\Psi}^{(s)}(w) \leqslant \Phi^{(s)}(w) \leqslant k|Q|^2 \tilde{\Psi}^{(s)}(w),$$

implying that the functions $\tilde{\Psi}^{(s)}$ and $\Phi^{(s)}$ define the same cost function. In particular, $\Phi^{(s)}$ is definable in cost MSO. □

## 6 Conclusions

We showed the decidability of the stochastic control problem. Our approach uses well quasi orders and the sequential flow problem, which is then solved using the theory of regular cost functions.

Together with the original result of [3,4] in the adversarial setting, our result contributes to the theoretical foundations of parameterised control. We return to the first application of this model, control of biological systems. As we discussed the stochastic setting is perhaps more satisfactory than the adversarial one, although as we saw very complicated behaviours emerge in the stochastic setting involving single agents, which are arguably not pertinent for modelling biological systems.

We thus pose two open questions. The first is to settle the complexity status of the stochastic control problem. Very recently [18] proved the **EXPTIME**-hardness of the problem, which is interesting because the underlying phenomena involved in this hardness result are specific to the stochastic setting (and do not apply to the adversarial setting). Our algorithm does not even yield elementary upper bounds, leaving a very large complexity gap. The second question is towards more accurately modelling biological systems: can we refine the stochastic control problem by taking into account the synchronising time of the controller, and restrict it to reasonable bounds?

## Acknowledgements

We thank Nathalie Bertrand and Blaise Genest for introducing us to this fascinating problem, and the preliminary discussions at the Simons Institute for the Theory of Computing in Fall 2015.
